# Effects of Stand Spatial Structure on Soil Enzyme Activities in Chinese Fir Plantations in the Mid-Subtropical Zone of China

**DOI:** 10.3390/plants15152336

**Published:** 2026-07-29

**Authors:** Han Zhang, Xiaoyu Cao, Wende Yan, Liu He, Xinhua Huang, Yuxin Xiang, Duoyi Zhang, Manni Hu, Hongrui Du, Shuqin Yang, Zhuohui Gao

**Affiliations:** 1 Faculty of Forestry, Central South University of Forestry and Technology, Changsha 410004, China; 20241100087@csuft.edu.cn (H.Z.); 20241200147@csuft.edu.cn (L.H.); xinhua_huang01@163.com (X.H.); 20231200113@csuft.edu.cn (Y.X.); 20251100030@csuft.edu.cn (D.Z.); 20251100066@csuft.edu.cn (M.H.); 20251200082@csuft.edu.cn (H.D.); 20251200081@csuft.edu.cn (S.Y.); 20251200098@csuft.edu.cn (Z.G.); 2 Key Laboratory of State Forestry Administration on Forest Resources Management and Monitoring in Southern Area, Changsha 410004, China; 3 National Engineering Laboratory of Applied Technology for Forestry and Ecology in Southern China, Changsha 410004, China; chongdelou716@csuft.edu.cn

**Keywords:** Chinese fir plantations, stand spatial structure, soil enzyme activities, redundancy analysis, response surface analysis, ecosystem functioning

## Abstract

Soil enzyme activities regulate organic matter decomposition and nutrient cycling and are widely used as indicators of microbial functioning and ecosystem processes. However, the mechanisms underlying their spatial heterogeneity, particularly the role of forest stand spatial structure, remain poorly understood. This study investigated subtropical Chinese fir (*Cunninghamia lanceolata*) plantations in southern China across different stand developmental stages. Relationships between stand spatial structure attributes and soil enzyme activities were evaluated using Spearman rank correlation analysis, redundancy analysis, and response surface methodology. Soil enzyme activities exhibited distinct stage-dependent variation, with generally lower activities in middle-aged stands and partial recovery in mature stands. The first two axes of redundancy analysis explained 62.96% of the variation in soil enzyme activities, while Spearman analysis showed significant negative associations between the complete mingling index and urease, catalase, and polyphenol oxidase activities (*r_s_* = −0.92 to −0.82, *p* < 0.01), whereas the competition index was positively associated with cellobiohydrolase activity (*r_s_* = 0.77, *p* < 0.05). Moreover, mingling and competition exhibited nonlinear and interactive effects on certain enzyme activities. These findings highlight the importance of stand spatial structure in regulating soil biochemical processes and provide ecological support for structure-oriented management of subtropical Chinese fir plantations.

## 1. Introduction

Soil enzymes are key drivers of biogeochemical functioning in forest ecosystems, facilitating the breakdown of organic matter and the cycling of essential nutrients such as carbon, nitrogen, and phosphorus [1]. Soil microorganisms and plant root systems represent the principal sources of these enzymes. These extracellular enzymes participate in organic matter decomposition, nutrient mineralization, and microbial resource acquisition processes in terrestrial ecosystems [2]. The enzyme groups examined in this study were selected because they represent major functional pathways associated with soil carbon, nitrogen, and phosphorus cycling, including C-, N-, and P-acquiring enzymes, as well as oxidative enzymes associated with organic matter transformation and microbial oxidative processes [3,4]. The rapid sensitivity of soil enzyme activities to variations in substrate supply and environmental conditions makes them effective indicators of changes in soil microbial functioning and associated ecosystem processes in plantation forests [5,6]. Therefore, maintaining stable soil enzyme activity is crucial for sustaining nutrient cycling and overall ecosystem functioning in plantation forests [7].

Chinese fir (*Cunninghamia lanceolata* (Lamb.) Hook.) is one of the most significant plantation tree species in subtropical China, extensively planted due to its rapid growth and high timber value [8,9]. However, the long-term management of Chinese fir plantations as pure and even-aged stands has led to the gradual degradation of soil quality. Such soil degradation is reflected in reductions in organic matter content, nutrient availability, and microbial activity, and these negative effects accumulate over successive rotation cycles [10,11]. Previous studies have primarily focused on changes in soil enzyme activities in Chinese fir plantations along stand age gradients. These studies have reported considerable variation in response patterns, ranging from continuous increases with stand development to distinct non-linear or stage-specific changes [12,13]. Although these studies suggest that stand development can influence soil biochemical processes, stand age alone appears insufficient to explain the high spatial variability of soil enzyme activity in Chinese fir plantations [14,15].

An increasing body of evidence indicates that, compared with stand age, forest stand spatial structure often plays a more prominent role in regulating belowground microbial processes in forest ecosystems [16]. A forest stand refers to a relatively homogeneous group of trees that share similar characteristics in species composition, age, structure, and growing conditions, and represents a fundamental unit for forest management and ecological research [17]. Stand spatial structure, including neighborhood composition, tree size distribution, and competitive interactions, can influence belowground microbial processes and related ecosystem functions by modifying the microclimatic environment, litter characteristics, and root inputs [18,19]. These structural attributes further affect soil enzyme activities by regulating the spatial heterogeneity of microbial habitats and substrate availability [20]. Previous studies have provided quantitative evidence that differences in forest structure and composition can substantially alter soil microbial properties and enzyme activities. For example, Farooq et al. (2022) demonstrated that forest type and soil depth significantly influenced soil enzymatic activities $\left(p\;<\;0.001\right)$, with higher microbial biomass carbon and nitrogen observed in mixed forests than in coniferous monoculture plantations [21]. Similarly, Wang et al. (2023) reported that in subtropical *Castanopsis hystrix* forests, mixed stands exhibited higher soil organic carbon, total nitrogen, microbial biomass carbon, and microbial biomass nitrogen than pure stands, accompanied by enhanced soil enzyme activities [22]. In structurally diverse or mixed forest stands, complementary litter properties and a broader range of resource inputs can promote microbial activity in soils and intensify enzymatic functioning [22,23]. In contrast, in stands with simplified spatial structures and intense tree competition, nutrient return to the soil may be constrained, thereby suppressing soil microbial functioning [21,24]. Despite the recognized importance of stand spatial structure in regulating ecological processes, its effects on soil enzyme activity in Chinese fir plantations remain insufficiently quantified and poorly understood.

Stand spatial structure can be quantified using various structural indices. In particular, the mingling index represents local tree species mixture [25], while the competition index estimates resource competition among neighboring trees [26,27]. Variations in these structural attributes can influence soil moisture conditions, litter decomposition processes, and microbial nutrient demand, thereby influencing soil enzyme activities involved in carbon (C), nitrogen (N), and phosphorus (P) cycling [28,29]. Accordingly, elucidating how species mingling and competitive intensity influence soil enzyme activity is essential for advancing our understanding of ecological processes in plantation forests and for guiding their sustainable management.

This research was carried out in Chinese fir plantations representing different developmental stages (young, middle-aged, and mature stands) within the subtropical monsoon zone of southern China. These developmental stages represent distinct phases of stand development. Young stands are characterized by rapid tree growth and structural development, middle-aged stands generally experience intensified competition and canopy closure, and mature stands generally exhibit more stabilized stand structure and resource availability [17]. The objectives were to: (1) quantify variations in stand spatial structure and soil enzyme activities across different stand age classes of Chinese fir plantations, and identify stage-specific differences during stand development; (2) identify stand spatial structure factors closely associated with soil enzyme activities, with particular emphasis on evaluating the effects of the complete mingling index and competition index on various soil enzymes and exploring their underlying mechanisms; and (3) assess the role of optimizing stand spatial structure for maintaining soil ecological functions in forest management, providing a basis for enhancing soil enzyme activity and promoting sustainable plantation management through regulation of species mixing and stand density.

## 2. Results

### 2.1. Spatial Structure Analysis

As shown in Table 1, the uniform angle index ranged from 0.54 to 0.59 across the three age classes, and did not differ significantly among them $\left(p\;>\;0.05\right)$, indicating a random spatial distribution pattern and relatively stable horizontal spatial arrangement. The competition index values in young, middle-aged, and mature stands were 5.98, 6.02, and 3.29, respectively. Young and middle-aged stands did not differ significantly $\left(p\;>\;0.05\right)$, but both showed significantly higher competition index values than mature stands $\left(p\;<\;0.05\right)$. This pattern may reflect stronger competitive interactions during the young and middle-aged stages, which remained relatively stable between these two developmental stages. In mature stands, the lower competition intensity may be associated with changes in stand density and structural development. The forest layer index values in young, middle-aged, and mature stands were 0.15, 0.21, and 0.15, respectively, with no significant differences among age classes $\left(p\;>\;0.05\right)$. Overall, the forest layer index remained consistently low, indicating limited vertical structural differentiation within the stands. More than 79–85% of trees were located in the same vertical layer as their neighboring trees, suggesting a low degree of vertical stratification within the stands. The complete mingling index values in young, middle-aged, and mature stands were 0.02, 0.10, and 0.04, respectively. The complete mingling index in middle-aged stands was significantly higher than that in young stands $\left(p\;<\;0.05\right)$, whereas no significant differences were observed between mature stands and the other two age classes $\left(p\;>\;0.05\right)$. Overall, the complete mingling index was close to the zero-mingling level, indicating a low degree of species mixing and limited spatial segregation among tree species, with most trees surrounded by individuals of the same species.

### 2.2. Soil Enzyme Activity Analysis

#### 2.2.1. Soil Enzyme Activities in the 0–45 cm Soil Layer Across Different Age Classes of Chinese Fir Plantations

Soil enzyme activities in the 0–45 cm soil layer across different age classes are shown in Table 2. Overall, soil enzyme activities exhibited distinct age-dependent patterns, with most enzymes showing an initial decline followed by partial recovery during stand development, whereas CBH activity continuously decreased with increasing stand age.

Soil URE activity in young, middle-aged, and mature stands was 0.397, 0.292, and 0.326 mg·g^−1^, respectively, decreasing initially and then increasing with stand age. ANOVA revealed significantly greater URE activity in young stands than in middle-aged and mature stands $\left(p\;<\;0.05\right)$. Soil ACP activity in young, middle-aged, and mature stands was 2.979, 2.866, and 3.310 mg·g^−1^, respectively, showing a similar decreasing–increasing trend. ACP activity was significantly greater in mature stands than in middle-aged stands $\left(p\;<\;0.05\right)$. Soil CAT activity in young, middle-aged, and mature stands was 3.001, 2.608, and 2.839 mL·g^−1^, respectively, also decreasing initially and then increasing with stand age. Soil INV activity in young, middle-aged, and mature stands was 16.197, 14.069, and 18.819 mg·g^−1^, respectively. INV activity declined at the middle-aged stage and increased in mature stands, with significantly higher values in mature than in middle-aged stands $\left(p\;<\;0.05\right)$. Soil PPO activity in young, middle-aged, and mature stands was 1.873, 1.332, and 1.558 mg·g^−1^, respectively, decreasing initially and then increasing with stand age. PPO activity was significantly greater in young than in middle-aged stands $\left(p\;<\;0.05\right)$. The overall reduction in enzyme activities observed during the middle-aged stage may indicate a temporary shift in soil biochemical functioning during stand development, potentially associated with increased resource competition and changes in soil biological processes.

In contrast, CBH activity declined steadily with stand age, with values of 0.628, 0.453, and 0.346 mg·g^−1^ in young, middle-aged, and mature stands, respectively. Young stands exhibited significantly higher CBH activity than the other two age classes $\left(p\;<\;0.05\right)$. The different response pattern of CBH compared with the other enzymes suggests that carbon acquisition processes may have distinct responses to stand development.

#### 2.2.2. Vertical Distribution of Soil Enzyme Activities Across Different Age Classes of Chinese Fir Plantations

**Figure 1:**
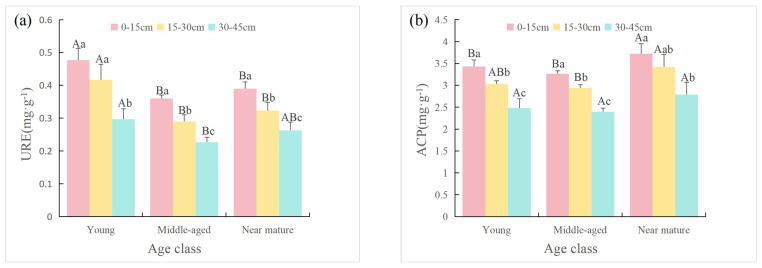

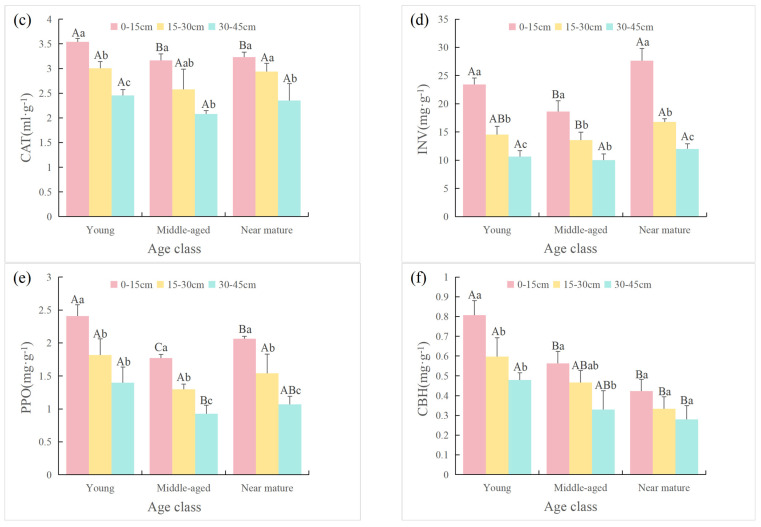
Variation in soil enzyme activities across soil layers and age classes, (**a**) URE, (**b**) ACP, (**c**) CAT, (**d**) INV, (**e**) PPO, and (**f**) CBH. Note: Significant differences among age classes within a given soil layer are indicated by different uppercase letters, while differences among soil layers within the same age class are represented by different lowercase letters (*p* < 0.05).

The vertical distribution of soil enzyme activities across age classes is presented in Figure 1, where panels (a–f) correspond to URE, ACP, CAT, INV, PPO, and CBH, respectively. Across all stand age classes, the activities of the six enzymes showed a consistent decreasing trend with increasing soil depth, with the highest activities generally occurring in the 0–15 cm soil layer. This pattern indicates that soil biochemical processes were mainly concentrated in surface soils.

Regarding stand age effects, URE, ACP, CAT, INV, and PPO generally exhibited a decline from young to middle-aged stands followed by partial recovery in mature stands across soil depths, whereas CBH showed a continuous decrease throughout stand development. The middle-aged stands generally exhibited lower enzyme activities, particularly in surface soils. In the 0–15 cm layer, which showed the strongest age-related responses, URE and CAT activities decreased significantly from young to middle-aged stands $\left(p\;<\;0.05\right)$, whereas INV and PPO activities declined from young to middle-aged stands and subsequently increased significantly from middle-aged to mature stands $\left(p\;<\;0.05\right)$. ACP activity also increased significantly from middle-aged to mature stands$\;\left(p\;<\;0.05\right)$, while CBH activity decreased significantly from young to middle-aged stands and continued to decline throughout stand development.

With increasing soil depth, age-related differences in enzyme activities became less pronounced. In the 15–30 cm layer, URE activity remained significantly higher in young stands than in middle-aged and mature stands $\left(p\;<\;0.05\right)$, whereas ACP and INV activities increased significantly from middle-aged to mature stands $\left(p\;<\;0.05\right)$, and CBH activity declined significantly from young to mature stands $\left(p\;<\;0.05\right)$. In the deepest soil layer (30–45 cm), URE and PPO activities decreased significantly from young to middle-aged stands, and CBH activity declined significantly from young to mature stands $\left(p\;<\;0.05\right)$, whereas ACP, CAT, and INV showed no significant differences among age classes.

The depth-related decline in enzyme activities was observed within each stand age class. Young stands showed significant depth-related variation in ACP, CAT, and INV activities $\left(p\;<\;0.05\right)$, while the other enzymes showed significant depth-related differences mainly between surface and deeper soil layers. Similar depth-dependent patterns were observed in middle-aged and mature stands, although the magnitude of variation differed among enzymes. These results suggest that soil depth played an important role in regulating enzyme activity distribution, with stronger enzymatic activity maintained in upper soil layers.

#### 2.2.3. Effects of Stand Age, Soil Depth, and Their Interaction on Soil Enzyme Activities

Two-way ANOVA (Table 3) showed that both stand age and soil depth had significant main effects on all six soil enzyme activities. Soil depth showed significant effects on all enzyme activities $\left(p\;<\;0.001\right)$, with relatively large F-values observed for INV $\left(F\;=\;184.995\right)$, PPO $\left(F\;=\;66.832\right)$, URE $\left(F\;=\;63.367\right)$, and ACP $\left(F\;=\;58.253\right)$, suggesting considerable variation in enzyme activities among soil layers.

Stand age also significantly affected all enzyme activities, with significant effects detected for URE, ACP, INV, PPO, and CBH $\left(p\;<\;0.001\right)$ and CAT $\left(p\;=\;0.003\right)$. Among the evaluated enzymes, relatively large F-values for CBH $\left(F\;=\;36.836\right)$, URE $\left(F\;=\;33.411\right)$, and INV $\left(F\;=\;26.442\right)$ reflected relatively strong statistical differences among stand age classes. These results further support the observed age-related variation patterns of soil enzyme activities.

The interaction between stand age and soil depth was significant only for INV activity $\left(F\;=\;5.605,\;p\;=\;0.004\right)$, suggesting that the response of INV to stand development varied among soil layers. In contrast, no significant interactions were observed for URE, ACP, CAT, PPO, or CBH $\left(p\;>\;0.05\right)$, indicating that no significant interaction between stand age and soil depth was detected for these enzymes.

### 2.3. Relationships Between Stand Spatial Structure and Soil Enzyme Activities

#### 2.3.1. Univariate Correlation Analysis Between Stand Spatial Structure and Soil Enzyme Activities

**Figure 2:**
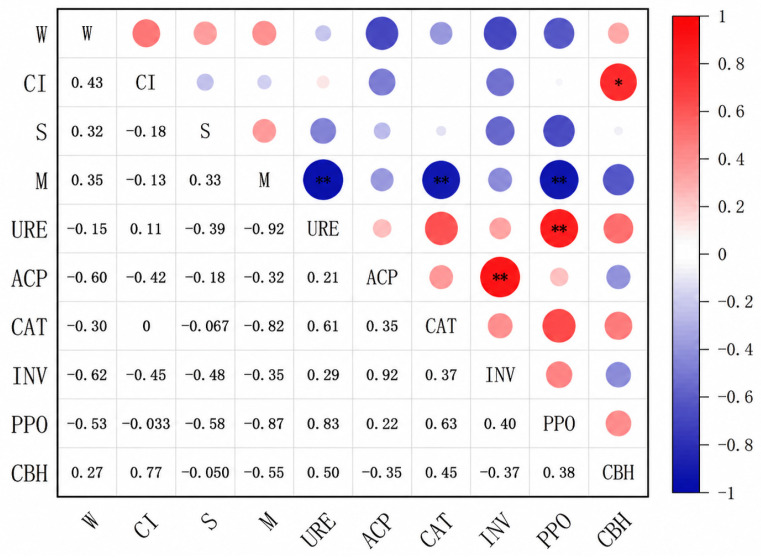
Heatmap of Spearman correlation coefficients between stand spatial structure indices and soil enzyme activities. * *p* < 0.05; ** *p* < 0.01. W, uniform angle index; CI, competition index; S, forest layer index; M, complete mingling index; URE, urease; ACP, acid phosphatase; CAT, catalase; INV, invertase; PPO, polyphenol oxidase; CBH, cellobiohydrolase.

The Spearman correlation matrix between stand spatial structure indices and soil enzyme activities is presented in Figure 2. The matrix illustrates the relationships between structural indices and enzyme activities, with red and blue colors representing positive and negative correlations, respectively, and darker colors indicating stronger correlations. The numbers in the matrix represent Spearman correlation coefficients $\left(r_{s}\right)$.

The correlation analysis showed that stand spatial structure indices exhibited different relationships with soil enzyme activities. The complete mingling index (M) showed the strongest correlations with several enzymes, exhibiting significant negative correlations with URE, CAT, and PPO activities $\left(p\;<\;0.01\right)$, with correlation coefficients of −0.92, −0.82, and −0.87, respectively. In contrast, the competition index (CI) showed a significant positive correlation with CBH activity $\left(r_{s}\;=\;0.77,\;p\;<\;0.05\right)$. No significant correlations were observed between ACP or INV activities and the evaluated structural indices.

These results suggest that species mingling and inter-tree competition may be associated with soil enzyme activities through different ecological processes, with the complete mingling index showing particularly strong relationships with several enzyme activities.

#### 2.3.2. Redundancy Analysis (RDA) of Stand Spatial Structure and Soil Enzyme Activities

**Figure 3:**
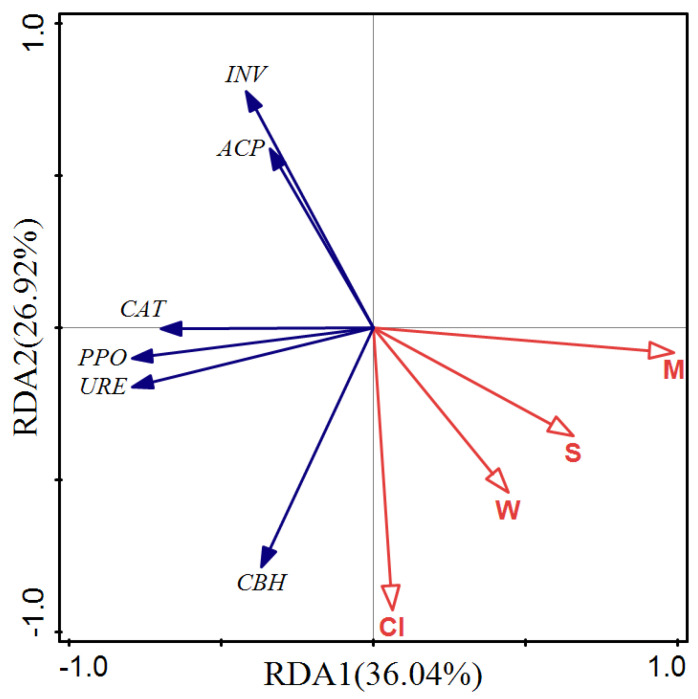
RDA of relationships between stand spatial structure indices and soil enzyme activities. W, uniform angle index; M, complete mingling index; S, forest layer index; CI, competition index; URE, urease; ACP, acid phosphatase; CAT, catalase; INV, invertase; PPO, polyphenol oxidase; CBH, cellobiohydrolase.

Redundancy analysis (RDA) is a constrained ordination method used to evaluate the linear relationships between explanatory variables and response variables. In this study, stand spatial structure indices were treated as explanatory variables, whereas soil enzyme activities were considered response variables. In the RDA ordination plot (Figure 3), the relationships among variables are reflected by the directions of the vectors, while longer vectors indicate stronger associations with the ordination axes.

As shown in Figure 3, the first and second axes of the RDA ordination accounted for 36.04% and 26.92% of the total variation in soil enzyme activities, respectively, with the first two axes together explaining 62.96% of the cumulative variation. Forward selection analysis indicated that the complete mingling index and the competition index exhibited relatively high explanatory power for the variation in soil enzyme activities, among which the complete mingling index showed the highest contribution. Further Monte Carlo permutation tests, which evaluate the statistical significance of explanatory variables by comparing the observed relationships with randomly permuted datasets, revealed that only the complete mingling index reached a statistically significant level $\left(p\;<\;0.05\right)$ (Table S1), identifying it as the primary stand spatial structure factor associated with variation in soil enzyme activities, whereas the competition index, despite its relatively high explanatory contribution, did not pass the significance test and was therefore regarded as a potential explanatory factor rather than a statistically significant explanatory variable for soil enzyme activities.

#### 2.3.3. Response Surface Analysis of Stand Spatial Structure Effects on Soil Enzyme Activities

**Figure 4:**
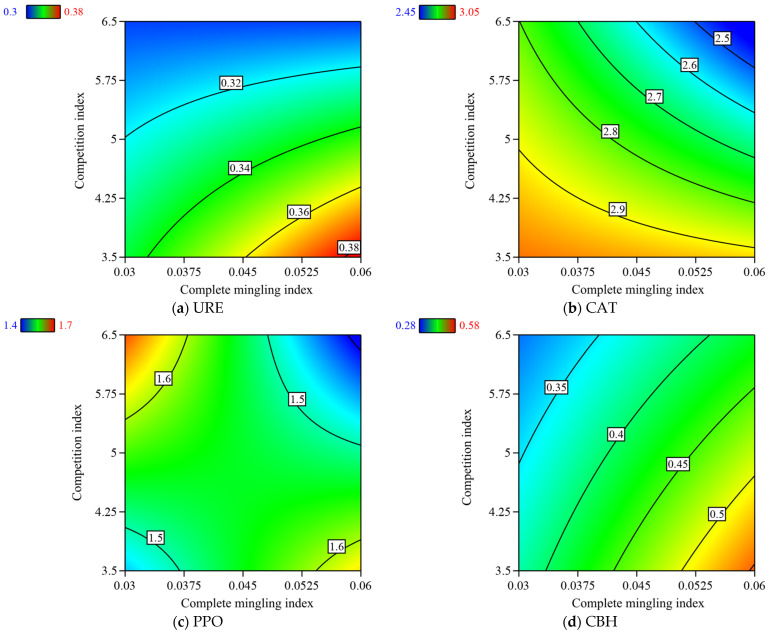
Contour plots of soil enzyme activities in relation to stand spatial structure indices based on response surface analysis. Note: Color gradients and contour lines represent the predicted enzyme activity values.

The above analyses indicated that the complete mingling index and the competition index were the key stand spatial structure indices closely associated with soil enzyme activities. Therefore, these two indices were selected as independent variables, and the activities of URE, CAT, PPO, and CBH were used as dependent variables for response surface analysis. A two-factor interaction (2FI) model was fitted to generate the corresponding contour plots (Figure 4) and regression equations (Table 4). The coefficients of determination $\left(R^{2}\right)$ for the response surface models of URE, CAT, and PPO activities were 0.984, 0.860, and 0.988, respectively, with adjusted $R^{2}$ values of 0.963, 0.787, and 0.971, indicating that the response surface models provided good fits for describing the relationships between stand spatial structure factors and soil enzyme activities. In contrast, the $R^{2}$ and adjusted $R^{2}$ values for the CBH activity model were 0.595 and 0.453, respectively, suggesting a moderate model fit; however, the model could still provide insights into the potential interactive effects of stand spatial structure factors on CBH activity.

Based on the regression equations for URE, CAT, PPO, and CBH activities (Table 4), the complete mingling index generally showed stronger associations with enzyme activities than the competition index in the constructed response surface models. The contour plot for URE activity (Figure 4a) showed that when the competition index was low, increasing the degree of mingling was associated with increased predicted URE activity. However, when the competition index reached medium to high levels, the positive association between stand mingling and URE activity was weakened, suggesting an interaction between the two factors. The contour plot for CAT activity (Figure 4b) indicated that CAT activity was highest when both the degree of mingling and the competition index were low, and decreased as either factor increased, suggesting that higher levels of these structural factors were associated with lower predicted CAT activity. The contour plot for PPO activity (Figure 4c) showed that, at a fixed level of competition, increasing the degree of mingling was associated with higher predicted PPO activity; similarly, increasing the competition index under a fixed level of mingling was also associated with higher predicted PPO activity. However, when both factors increased simultaneously, PPO activity decreased, indicating a negative interaction between stand mingling and the competition index. The contour plot for CBH activity (Figure 4d) showed that at low levels of competition, higher stand mingling was associated with higher CBH activity; however, as the competition index increased, CBH activity showed a decreasing trend.

## 3. Discussion

### 3.1. Stage-Dependent Variation in Soil Enzyme Activities During Stand Development

Soil enzyme activities in Chinese fir plantations exhibited clear stage-related variation during stand development. Overall, enzyme activities were relatively high in young stands, declined markedly in middle-aged stands, and partially recovered in mature stands. Across the 0–45 cm soil layer, most measured enzymes, except CBH, reached their lowest levels in middle-aged stands, indicating that the middle-aged stage may represent a transitional period in soil biochemical functioning [14]. These results suggest that soil enzymatic functioning in plantation ecosystems may vary with stand developmental stages rather than following a simple linear trajectory.

Similar stage-dependent variations in soil biochemical processes have been reported in other forest ecosystems, suggesting that ecosystem development can regulate microbial activity, substrate availability, and nutrient demand [30,31]. However, most previous studies have focused mainly on stand age or ecosystem succession, whereas our results further suggest that different enzyme groups may exhibit distinct responses during Chinese fir plantation development.

During the middle-aged stage, rapid tree growth may increase plant demand for available nutrients, particularly nitrogen and phosphorus, which may intensify competition between plants and microorganisms for limited resources during this developmental phase [32]. At the same time, progressive canopy closure may alter understory vegetation dynamics, litter inputs, and forest-floor microclimatic conditions [33,34]. Such structural changes may modify the quantity and quality of organic substrate inputs, potentially affecting microbial resource availability and extracellular enzyme production [13,35].

The lower enzyme activities observed in middle-aged stands may therefore reflect a temporary adjustment of soil biochemical processes during rapid stand development, rather than a permanent decline in ecosystem functioning. This interpretation is consistent with the transitional characteristics commonly observed during intermediate stages of forest development [36].

As stands develop further into the mature stage, the stabilization of stand structure and increased litter accumulation commonly observed during later developmental stages of forest ecosystems may enhance substrate availability and support microbial recovery [37]. These changes could partly explain the rebound of several soil enzyme activities observed in this study. Similar stage-dependent changes in soil enzyme activities have also been reported in other forest ecosystems. For example, Allison et al. (2007) [38] found that soil enzyme activities changed systematically along a forest chronosequence, with phosphorus-acquiring enzymes showing strong positive relationships with ecosystem development $\left(R\;>\;0.90,\;p\;<\;0.001\right)$. These findings indicate that enzyme activities are closely linked to changes in resource availability during forest development, supporting our observation that soil enzymes exhibited distinct stage-dependent responses across Chinese fir plantations [38].

Compared with previous studies that mainly emphasized stand age effects on soil biochemical properties, our findings highlight that enzyme responses during stand development are enzyme-specific and may reflect different microbial resource acquisition strategies [31].

In contrast to the other enzymes examined, CBH activity declined continuously with stand age. This distinct pattern may indicate that carbon acquisition processes responded differently from enzymes associated with nutrient acquisition during stand development. Variations in litter composition and substrate availability may influence the decomposition of cellulose and other recalcitrant carbon compounds, thereby affecting enzymes involved in structural carbon degradation [39,40]. The continuous decline of CBH activity suggests that carbon-degrading enzymes may exhibit different developmental trajectories compared with enzymes involved in nitrogen and phosphorus acquisition [31].

These contrasting responses among enzyme groups highlight the functional differentiation of microbial strategies for resource acquisition and suggest that stand development may alter different components of soil biochemical functioning in different ways [41]. However, stand age alone may not fully explain the variation in soil enzyme activities, because forest development is also accompanied by changes in stand spatial characteristics, including species mingling, tree competition, and structural heterogeneity. Therefore, further evaluation of how stand spatial structure contributes to variations in soil enzyme activities is necessary to better understand the mechanisms underlying soil biochemical changes in Chinese fir plantations.

### 3.2. Effects of Stand Spatial Structure on Soil Enzyme Activities

Beyond stand developmental stage, variation in stand spatial structure also played an important role in influencing soil enzyme activities in Chinese fir plantations. Results from both Spearman correlation and redundancy analysis indicated that the complete mingling index and competition index explained substantially more variation in enzyme activities than the uniform angle index and forest layer index. This pattern suggests that structural attributes associated with species mixture and competitive interactions may have stronger relationships with soil biochemical processes than purely geometric aspects of stand spatial arrangement [29]. In other words, the functional characteristics of stand structure may be more important than spatial pattern alone in shaping soil biochemical processes.

Previous studies have demonstrated that tree species diversity and structural heterogeneity can influence below-ground ecosystem functioning by modifying resource distribution and species interactions [42]. However, many previous studies have focused on biodiversity or stand-level diversity effects, whereas our results provide further evidence that quantitative stand spatial structure indices can help explain variations in soil enzyme activities [42,43].

Stand spatial structure may influence soil enzyme activities through its potential effects on forest microenvironmental conditions and resource distribution [34]. The spatial arrangement of trees may modify canopy structure, light penetration, and litter deposition patterns, which can subsequently affect soil moisture conditions, organic matter inputs, and nutrient availability, thereby influencing microbial processes and enzyme activities [44,45]. In stands characterized by high density and strong competition, trees tend to dominate resource acquisition, potentially altering the transfer of carbon and nutrients to the soil through changes in litterfall and root turnover. Such changes may constrain microbial activity and associated enzymatic processes in the soil [46,47].

Among the structural indices evaluated in this study, the complete mingling index emerged as the most important factor explaining variation in soil enzyme activities. This result highlights the importance of tree species spatial configuration in shaping soil ecological processes. Higher levels of species mingling may increase heterogeneity in litter inputs and root distributions, thereby providing more diverse substrate resources for soil microorganisms [48]. Greater substrate diversity and resource complementarity have been suggested to promote microbial activity and enzymatic functioning in forest soils [49,50]. Compared with studies that have primarily examined the effects of tree species diversity on below-ground processes [42], our findings suggest that the spatial arrangement of neighboring trees may provide an additional perspective for understanding the relationship between forest structure and soil biochemical processes.

Although the competition index did not reach statistical significance in the Monte Carlo permutation test, it still showed notable explanatory power in the ordination analysis. One possible explanation is that Chinese fir plantations in the study area have been managed as relatively uniform and even-aged stands for a long period, resulting in relatively similar competitive environments among plots. Under such conditions, competition may be dominated by intraspecific interactions, which may reduce variability in competition intensity across plots and weaken its statistical detectability as an independent explanatory factor [16]. Nevertheless, competitive interactions remain an important component of stand structure and may still influence soil ecological processes through their effects on resource allocation and stand density. This result suggests that statistical significance and ecological importance do not always completely overlap, particularly when structural variables are strongly associated with each other or when environmental gradients are relatively limited [51].

Response surface analysis further revealed nonlinear and interactive relationships between the complete mingling index and competition index in regulating several soil enzyme activities. Under relatively low competition intensity, moderate increases in species mingling were associated with higher enzyme activities. However, when competition intensity increased, the positive effects of increased mingling became weaker or even shifted toward inhibitory responses. These results indicate that the effects of different structural attributes are not independent but may interact in shaping soil enzyme activities. The interactive effects revealed by response surface analysis further extend previous studies by demonstrating that stand spatial structure components may jointly regulate soil biochemical processes rather than acting independently [52].

From a forest management perspective, these findings highlight the ecological importance of optimizing stand spatial structure in Chinese fir plantations. In particular, the middle-aged stand stage may represent a critical period during which structural regulation may provide an opportunity to influence soil ecological functioning. Implementing moderate thinning to alleviate excessive competition, together with promoting appropriate levels of species mingling through mixed planting or underplanting, may improve stand structural heterogeneity and potentially support soil biochemical processes [48,53]. Such structure-oriented management strategies may enhance the long-term sustainability and ecological functioning of subtropical plantation forests.

## 4. Materials and Methods

### 4.1. Site Description

**Figure 5:**
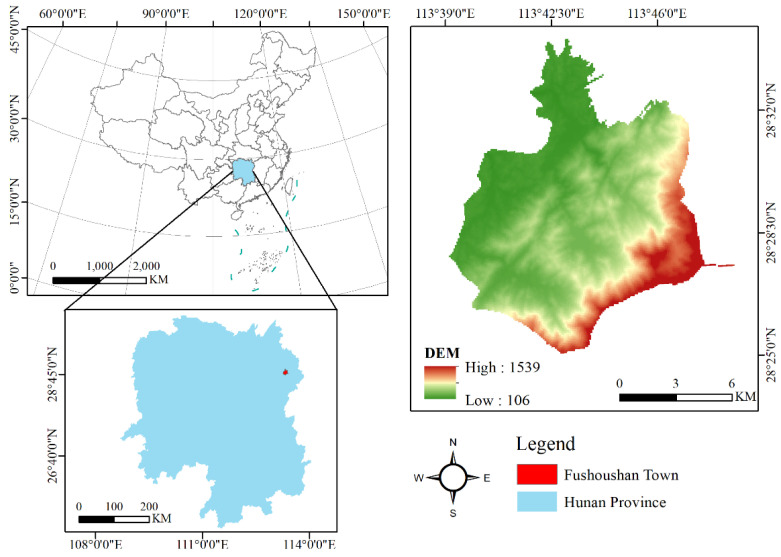
Location of the study area in Fushou Forest Farm, Pingjiang County, Hunan Province, China.

Field investigations were conducted at Fushou Forest Farm in Pingjiang County, Hunan Province, China (Figure 5). The study area is located on Mount Fushou in the southern part of the county (28°03′00″–28°32′30″ N, 113°41′15″–113°45′00″ E) and encompasses approximately 1274.9 ha. The region is characterized by a humid subtropical monsoon climate, with a mean annual temperature of 12.1 °C, average yearly precipitation of roughly 2100 mm, about 1500 h of sunshine per year, and a relative humidity averaging 87%. Elevation ranges from 835 to 1573.2 m above sea level, and the terrain is heterogeneous, characterized by steep slopes at lower elevations and gentler slopes at mid-elevations, which are conducive to forest growth. The dominant soil type in the study plots was mountain yellow-brown soil. Apart from natural secondary forests, all other forest types are plantations established on clear-cut sites. Since 2006, these plantations have been designated as public welfare forests and have received minimal management intervention, apart from occasional thinning for stand tending.

All Chinese fir trees in the study plots were plantation-grown. Young stands contained scattered associated tree species, including *Paulownia fortunei* (Seem.) Hemsl., *Pinus massoniana* Lamb., *Melia azedarach* L., and *Pinus taiwanensis* Hayata. Middle-aged stands contained sporadic associated tree species, mainly *Cryptomeria fortunei* Hooibrenk ex Otto & Dietr., *Prunus davidiana* (Carrière) Franch., *Robinia pseudoacacia* L., and *Magnolia officinalis* Rehder & E.H. Wilson. Mature stands contained scattered associated tree species, including *Phyllostachys heterocycla* (Carrière) Mitford, *Prunus tomentosa* Thunb., *Quercus fabri* Hance, and *Betula luminifera* H.J.P. Winkl. During stand development, tending thinning operations were conducted once in middle-aged stands and twice in mature stands, whereas no thinning operations had been carried out in young stands.

### 4.2. Experimental Design

Following a comprehensive field survey of representative stands within the forest farm, thirty fixed plots (20 m × 30 m) were established in Chinese fir plantations under comparable site conditions. Each plot was subdivided into six 10 m × 10 m subplots, which were used as sampling units for tree measurements. Within each subplot, all living trees were surveyed, and data on species identity, diameter at breast height (DBH), and tree height were collected (Table 5). A total of 4784 trees were measured across the 30 fixed plots, including 1690 trees in young stands, 1657 trees in middle-aged stands, and 1437 trees in mature stands.

**Figure 6:**
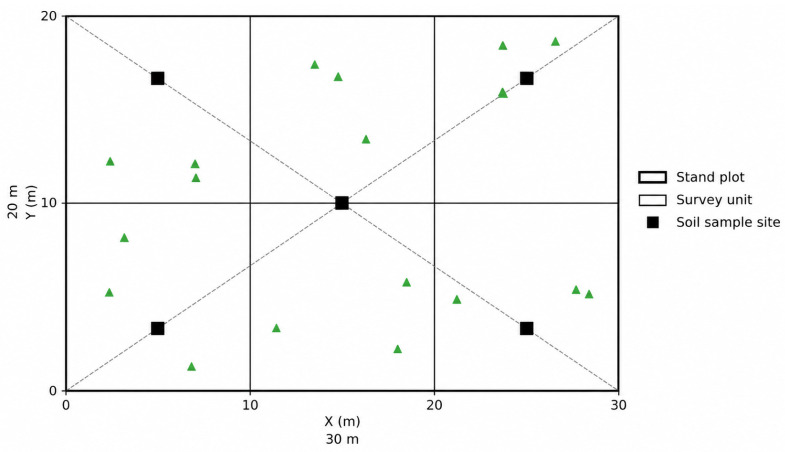
Schematic diagram of soil sampling design. Green triangles represent schematic illustrations of trees within the stand and do not indicate the actual spatial distribution of individual trees.

Soil samples were collected during the summer growing season (June–August) in subtropical Chinese fir plantations. For soil sampling, the established plots were grouped into three stand age classes based on stand developmental stage (young, middle-aged, and mature), with ten replicate plots assigned to each stand age class. Soil sampling within each plot was performed at five locations positioned in an X-shaped configuration (Figure 6) across three depth intervals (0–15 cm, 15–30 cm, and 30–45 cm). For every depth layer, subsamples obtained from the five locations in a given plot were pooled to produce a single composite sample, resulting in ten replicated composite samples per soil layer for each stand age class. Altogether, ninety soil samples (approximately 1 kg per sample) were obtained. Following collection, samples were maintained at 4 °C and transported to the laboratory for subsequent determination of soil enzyme activities.

### 4.3. Selection and Calculation of Stand Spatial Structure Indices

Stand spatial structure indices are quantitative measures that describe spatial relationships among trees within a stand, including species identity, tree size, and horizontal and vertical distribution patterns. The complete mingling index provides an integrated measure of local species mixing by accounting for the focal tree and the species relationships among its neighboring trees [54]. The uniform angle index was applied to describe the horizontal distribution pattern of individual trees [55], whereas the forest layer index was used to characterize the vertical structural differentiation of stands [56]. The competition index was employed to quantify the intensity of resource competition among neighboring trees [57]. The analysis of stand spatial structure incorporated four indices: the complete mingling, uniform angle, forest layer, and competition indices. A neighborhood size of four trees (n = 4) was adopted for index calculations. To reduce edge-related bias from trees located beyond plot boundaries, each plot was surrounded by a 3 m buffer zone. The formulas for calculating these indices are presented below [58,59].

(1) Uniform angle index:

(1) $$W_{i}=\frac{1}{n}\sum_{j=1}^{n}Z_{\mathrm{ij}}$$

where $W_{i}$ is the uniform angle index of the reference tree *i*. If the angle formed by two adjacent trees is smaller than the standard angle, $Z_{ij}\;=\;1$; otherwise, $Z_{ij}\;=\;0$. The standard angle is defined as $360{}^{\circ}/\left(n\;+\;1\right)$. When four neighboring trees are considered, the possible values of the uniform angle index $W_{i}$ are 0, 0.25, 0.50, 0.75, and 1, which represent very regular, regular, random, irregular, and very irregular spatial distribution patterns of trees within a structural unit, respectively [55].

(2) Complete mingling index:

(2) $$M_{c_{i}}=\frac{1}{2}\left(D_{i}+\frac{c_{i}}{n_{i}}\right)\times M_{i}$$

where $M_{c_{i}}$ is the complete mingling index of the reference tree *i*. $D_{i}$ represents the Simpson index of the structural unit in which the reference tree *i* is located; $M_{i}$ is the simple mingling index of the reference tree *i*; $n_{i}$ denotes the number of neighboring trees within the structural unit of the reference tree *i*; and $c_{i}$ is the number of different tree species among the neighboring trees. The value of $M_{c_{i}}$ ranges from 0 to 1 and was classified into five intervals: 0, (0, 0.25], (0.25, 0.5], (0.5, 0.75], and (0.75, 1], corresponding to zero mingling, weak mingling, moderate mingling, strong mingling, and very strong mingling among trees, respectively.

(3) Forest layer index:

(3) $$S_{i}=\frac{z_{i}}{3}\times \frac{1}{n}\sum_{j=1}^{n}s_{\mathrm{ij}}$$

where $S_{i}$ is the forest layer index of the reference tree *i*. $z_{i}$ denotes the number of vertical layers within the spatial structural unit of the reference tree *i*. $s_{ij}$ is a binary variable. When the reference tree *i* and its *j*th neighboring tree belong to different vertical layers, $s_{ij}\;=\;1$; when they occur in the same layer, $s_{ij}\;=\;0$ The value of $S_{i}$ ranges from 0 to 1. Values of the forest layer index closer to 1 indicate a more complex vertical stand structure.

(4) Competition index:

(4) $$CI_{i}=\sum_{j=1}^{n}\left(\frac{D_{j}}{D_{i}}\right)\times \frac{1}{L_{\mathrm{ij}}}$$

where ${CI}_{i}$ is the competition index of the reference tree *i*. $D_{i}$ and $D_{j}$ denote the diameters at breast height of the reference tree *i* and the *j*th neighboring tree, respectively. $L_{ij}$ represents the horizontal distance between the reference tree *i* and the neighboring tree *j*. *n* denotes the number of neighboring trees within the structural unit of the reference tree *i*. This index reflects the competitive pressure exerted by neighboring trees on the reference tree. Higher values indicate stronger competition intensity.

### 4.4. Determination of Soil Enzyme Activities

Soil enzyme activities were determined using established colorimetric, titrimetric, and fluorometric methods. The activities of six enzymes associated with soil carbon, nitrogen, and phosphorus cycling, including urease (URE), acid phosphatase (ACP), catalase (CAT), invertase (INV), polyphenol oxidase (PPO), and cellobiohydrolase (CBH), were measured. Colorimetric assays were performed using a UV–visible spectrophotometer (Shanghai INESA Analytical Instrument Co., Ltd., Shanghai, China), whereas fluorometric assays were performed using a fluorescence microplate reader (Allsheng Instruments Co., Ltd., Hangzhou, China). Titrimetric assays were conducted using standard volumetric procedures following the method described by Alef and Nannipieri [60].

#### 4.4.1. Hydrolytic Enzyme Activities

Soil urease (URE) activity was determined using the indophenol blue colorimetric method. Urease activity was quantified based on the amount of NH_4_^+^ released during urea hydrolysis. The extracted ammonium was reacted with color-developing reagents to form indophenol blue, and the absorbance was measured spectrophotometrically at 630 nm. Enzyme activity was calculated according to the concentration of NH_4_^+^ released from urea hydrolysis and expressed on a dry soil basis [61].

Soil acid phosphatase (ACP) activity was determined using p-nitrophenyl phosphate (pNPP) as the substrate. Soil samples were incubated with pNPP in modified universal buffer under acidic conditions. After termination of the reaction with CaCl_2_ and NaOH, the released p-nitrophenol (pNP) was quantified colorimetrically at 410 nm using a pNP standard curve. ACP activity was calculated based on the amount of pNP released and expressed per gram of dry soil per hour [62].

Soil invertase (INV) activity was determined based on the hydrolysis of sucrose to reducing sugars. Soil samples were incubated with sucrose as the substrate, and the released reducing sugars were quantified using the 3,5-dinitrosalicylic acid (DNS) colorimetric method. The concentration of reducing sugars was measured at 540 nm using glucose as the standard. Invertase activity was calculated based on the amount of glucose equivalents produced and expressed on a dry soil basis [63].

Soil cellobiohydrolase (CBH) activity was determined using a fluorometric microplate assay with 4-methylumbelliferyl (4-MUF)-linked β-D-cellobioside as the substrate. Soil suspensions were incubated with the fluorescent substrate in microplates under dark conditions. CBH activity was quantified based on the release of 4-methylumbelliferone (4-MUF), and fluorescence intensity was measured using a microplate fluorometer at 365 nm excitation and 450 nm emission. Enzyme activity was calculated based on the amount of 4-MUF released [64].

#### 4.4.2. Oxidative Enzyme Activities

Soil catalase (CAT) activity was determined using the potassium permanganate titration method. The enzyme activity was measured based on the decomposition of hydrogen peroxide (H_2_O_2_). After incubation of soil samples with H_2_O_2_, the residual H_2_O_2_ was quantified by titration with KMnO_4_ after reaction termination with H_2_SO_4_. Catalase activity was calculated based on the amount of H_2_O_2_ decomposed after blank correction and expressed on a dry soil basis [60].

Soil polyphenol oxidase (PPO) activity was measured using the L-DOPA spectrophotometric method. Soil suspensions were incubated with L-3,4-dihydroxyphenylalanine (L-DOPA) as the substrate under appropriate buffer conditions. The formation of oxidation products was quantified spectrophotometrically, and PPO activity was calculated from the increase in absorbance over time. Absorbance was measured at 460 nm, and enzyme activity was expressed based on the rate of substrate oxidation [65].

### 4.5. Statistical Data Analysis

One-way analysis of variance (ANOVA) followed by Tukey’s honestly significant difference (HSD) test was used to examine differences in stand spatial structure characteristics among Chinese fir plantations across different age classes. Two-way ANOVA followed by Tukey’s HSD test was applied to analyze the effects of stand age and soil depth on soil enzyme activities.

Associations between stand spatial structure and soil enzyme activities were examined using Spearman’s rank correlation analysis to evaluate the strength and direction of monotonic relationships among variables. The Spearman correlation coefficient $\left(r_{s}\right)$ was calculated as follows [61]:

(5) $$r_{s}(i,k)=1-\frac{6\sum_{j=1}^{N}\left(x_{ij}-{\bar{x}}_{i}\right){\left(x_{kj}-{\bar{x}}_{k}\right)}^{2}}{N^{3}-N}$$

where *N* is the total number of samples. $x_{ij}$ and $x_{kj}$ represent the ranks of indices *i* and *k* in sample *j*, respectively. ${\bar{x}}_{i}$ and ${\bar{x}}_{k}$ denote the mean ranks of indices *i* and *k* across all samples. The value of the Spearman correlation coefficient $r_{s}$ ranges from −1 to 1. When $r_{s}\;=\;1$, the two variables exhibit a perfect positive monotonic relationship; when $r_{s}\;=\;-1$, a perfect negative monotonic relationship is indicated; and when $r_{s}\;=\;0$, no monotonic relationship exists between the two variables.

Linear relationships between stand spatial structure and soil enzyme activities were evaluated using redundancy analysis (RDA). The choice of ordination method was guided by an initial detrended correspondence analysis (DCA). The results showed that the gradient length of the first ordination axis was 0.14, indicating that the data were predominantly linearly distributed; therefore, RDA was deemed suitable for subsequent analysis. The significance of individual explanatory variables in the ordination was evaluated using Monte Carlo permutation tests (999 permutations) [66].

Based on the key stand spatial structure factors identified above, response surface methodology (RSM) was applied to construct two-factor interaction (2FI) models. The 2FI model was selected based on model significance and adequacy criteria. The RSM framework was used to assess the interactive effects of key stand spatial structure factors on soil enzyme activities and to examine variation patterns under different combinations of spatial structural factors. The regression model was expressed as follows [67]:

(6) $$y(x)=\beta_{0}+\sum_{i=1}^{P}\beta_{i}X_{i}+\sum_{i=1}^{P}\sum_{j=i+1}^{P}\beta_{\mathrm{ij}}X_{i}X_{j}+\varepsilon$$

where $X_{i}$ and y(x) represent the independent variables and the response variable, respectively;$\;\beta_{0}$, $\beta_{i}$ and $\beta_{ij}$ denote the intercept, linear effects and interaction effects, respectively; and *ε* represents the random error term.

In the above statistical analyses, one-way ANOVA, two-way ANOVA, and Spearman correlation analysis were performed using Microsoft Excel 2019 (Microsoft Corporation, Redmond, WA, USA) and SPSS 27.0 (IBM Corp., Armonk, NY, USA) for data analysis and graphical processing. RDA was conducted with CANOCO 5 (Microcomputer Power, Ithaca, NY, USA), and RSM was performed using Design-Expert 13 (Stat-Ease Inc., Minneapolis, MN, USA).

## 5. Conclusions

This study investigated subtropical Chinese fir plantations in southern China and examined patterns of soil enzyme activities from the perspective of stand spatial structure. The results showed that soil enzyme activities exhibited pronounced stage-dependent variation during stand development, and stand spatial structure played an important role in regulating soil enzymatic functioning. Among the evaluated structural indices, the complete mingling index showed stronger associations with variation in multiple soil enzymes, whereas the competition index influenced certain enzymes and showed nonlinear relationships within specific ranges. These findings indicate that structural attributes reflecting tree species spatial configuration and inter-tree competition can shape soil biochemical processes in Chinese fir plantations. In particular, the middle-aged stage appears to represent a period during which soil biochemical processes are especially sensitive to spatial structure regulation. Moderate thinning to alleviate competition and optimizing stand spatial configuration during this stage may help maintain higher soil enzyme activities and promote nutrient cycling. Overall, this study provides new insights into how stand spatial structure regulates soil biochemical processes and offers ecological support for structure-oriented management of subtropical plantation forests.

## Figures and Tables

**Table 1:** Variation in stand spatial structure indices among Chinese fir plantations of different age classes.

Stand Age Class	Uniform Angle Index	Competition Index	Forest Layer Index	Complete Mingling Index
Young	0.57 ± 0.03 a	5.98 ± 0.33 a	0.15 ± 0.01 a	0.02 ± 0.01 b
Middle-aged	0.59 ± 0.01 a	6.02 ± 0.93 a	0.21 ± 0.07 a	0.10 ± 0.04 a
Mature	0.54 ± 0.02 a	3.29 ± 0.23 b	0.15 ± 0.03 a	0.04 ± 0.01 ab

Note: Values sharing different lowercase letters within a column differ significantly among age classes (*p* < 0.05).

**Table 2:** Variation in soil enzyme activities within the 0–45 cm soil layer among Chinese fir plantations of different age classes.

Age Class	URE (mg·g^−1^)	ACP (mg·g^−1^)	CAT (mL·g^−1^)	INV (mg·g^−1^)	PPO (mg·g^−1^)	CBH (mg·g^−1^)
Young	0.397 ± 0.032 a	2.979 ± 0.136 ab	3.001 ± 0.093 a	16.197 ± 0.989 ab	1.873 ± 0.214 a	0.628 ± 0.066 a
Middle-aged	0.292 ± 0.01 b	2.866 ± 0.051 b	2.608 ± 0.158 a	14.069 ± 1.356 b	1.332 ± 0.053 b	0.453 ± 0.072 b
Mature	0.326 ± 0.02 b	3.31 ± 0.248 a	2.839 ± 0.204 a	18.819 ± 1.175 a	1.558 ± 0.122 ab	0.346 ± 0.063 b

Note: Values sharing different lowercase letters within a column differ significantly among age classes (*p* < 0.05).

**Table 3:** Variation in the effects of stand age, soil depth, and their interaction on soil enzyme activities.

Soil Enzyme Activity	Stand Age		Soil Depth		Stand Age × Soil Depth	
	F	*p*	F	*p*	F	*p*
URE	33.411	<0.001	63.367	<0.001	1.309	0.304
ACP	14.412	<0.001	58.253	<0.001	0.107	0.978
CAT	8.156	0.003	54.026	<0.001	0.474	0.754
INV	26.442	<0.001	184.995	<0.001	5.605	0.004
PPO	21.842	<0.001	66.832	<0.001	0.236	0.914
CBH	36.836	<0.001	25.092	<0.001	1.498	0.245

Note: Data were analyzed using two-way ANOVA with stand age and soil depth treated as fixed factors. Differences were considered significant at (*p* < 0.05).

**Table 4:** Response surface regression equations for soil enzyme activities in relation to stand spatial structure indices.

Independent Variables	Response Surface Regression Equation	*R* ^2^
Complete mingling index and competition index	$Y_{1}=0.267+3.467X_{1}+0.006X_{2}-0.534X_{1}X_{2}$	0.984
	$Y_{2}=2.856+11.283X_{1}+0.054X_{2}-3.810X_{1}X_{2}$	0.860
	$Y_{3}=0.486+23.963X_{1}+0.229X_{2}-5.199X_{1}X_{2}$	0.988
	$Y_{4}=0.206+8.403X_{1}+0.0002X_{2}-0.747X_{1}X_{2}$	0.595

Note: *Y*_1_, *Y*_2_, *Y*_3_, and *Y*_4_ represent urease, catalase, polyphenol oxidase, and cellobiohydrolase activity, respectively. *X*_1_ represents the complete mingling index, and *X*_2_ represents the competition index.

**Table 5:** General description of the sample plots.

Stand Age Class	Stand Age	Stand Density	Mean Tree Height	Mean DBH	Mean Slope	Aspect	Mean Elevation	Soil Type	Number of Thinning Events
	(yr)	(Trees·ha^−1^)	(m)	(cm)	(°)		(m)		
Young	6	2817	2.6	2.7	21	North, northwest	958	Yellow-brown soil	0
Middle-aged	13	2761	5.7	8.9	19	North	976	Yellow-brown soil	1
Mature	25	2395	8.9	12.5	22	North, northwest	962	Yellow-brown soil	2

Note: Values represent the mean of 10 replicate plots for each stand age class (n = 10). DBH was measured at 1.3 m above ground level. All middle-aged stand plots were located on north-facing slopes, whereas young and mature stand plots were located on north- or northwest-facing slopes.

## Data Availability

The data that support the findings of this study are available from the corresponding author upon reasonable request.

## Supplementary Materials

The following supporting information can be downloaded at: https://www.mdpi.com/article/10.3390/plants15152336/s1, Table S1: Results of Monte Carlo permutation tests for the effects of stand spatial structure on soil enzyme activities.
