# Supplementary material for: Effects of Stand Spatial Structure on Soil Enzyme Activities in Chinese Fir Plantations in the Mid-Subtropical Zone of China
Source: Plants (Basel). 2026 Jul 29;15(15):2336. doi: 10.3390/plants15152336 (PMC13468024; doi:10.3390/plants15152336)
Supplement: Supplementary file 1 [file plants-15-02336-s001.zip › plants-4417157-supplementary.pdf]

## Supplementary Material

# Effects of Stand Spatial Structure on Soil Enzyme Activities in Chinese Fir Plantations in the Mid-Subtropical Zone of China

**This file contains supplementary information supporting the findings of this study.**

### A. Supplementary tables

Table S1.**Supplementary table**

**Table S1.** Results of Monte Carlo permutation tests for the effects of stand spatial structure on soil enzyme activities.

| Soil enzyme activities | | | |
|---|---|---|---|
| Variable | Contribution (%) | pseudo-F | *p* Value |
| W | 16.1 | 2.1 | 0.141 |
| CI | 18.4 | 2.3 | 0.112 |
| S | 10.9 | 1.4 | 0.289 |
| M | 23.3 | 3.0 | **0.046*** |

* Indicates statistical significance at $p < 0.05$. W, uniform angle index; CI, competition index; S, forest layer index; M, complete mingling index. *p* values were obtained using Monte Carlo permutation tests with 999 permutations.
